# *Notes from the Field*: Powassan Virus Disease in an Infant — Connecticut, 2016

**DOI:** 10.15585/mmwr.mm6615a3

**Published:** 2017-04-21

**Authors:** Jessica W. Tutolo, J. Erin Staples, Lynn Sosa, Nicholas Bennett

**Affiliations:** ^1^Division of Pediatric Infectious Diseases and Immunology, Connecticut Children's Medical Center, Hartford, Connecticut; ^2^Division of Vector-Borne Diseases, CDC, Fort Collins, Colorado; ^3^Connecticut Department of Public Health.

In early November 2016, a previously healthy male infant aged 5 months from eastern Connecticut developed fever and vomiting. Right-sided facial twitching began over the next several days, and progressed to seizures that included rightward eye deviation and right arm stiffening. He was admitted to the hospital for evaluation and management of his seizures. There was no travel history; however, the parents reported that 2 weeks earlier, the infant had been bitten by a tick most likely carried into the home on a family member’s clothing. The estimated time of tick attachment was <3 hours.

Head computed tomography scan, complete blood count, and serum electrolytes were normal. A lumbar puncture was performed; white blood cell count in the cerebrospinal fluid (CSF) was 125/*μ*L (81% lymphocytes) (reference range = 0–15 cells/*μ*L). Magnetic resonance imaging (MRI) of the brain showed a symmetric pattern of restricted diffusion (suggestive of cellular edema) involving the basal ganglia, rostral thalami, and left pulvinar, consistent with encephalitis. There were no findings of hemorrhage, and the lesions did not enhance, indicating normal local blood flow and minimal inflammation. Testing for common nonarboviral causes of encephalitis was negative, as were CSF bacterial cultures and respiratory viral cultures.

Because of the clinical and MRI findings, and the confirmed brief tick attachment, CDC testing for evidence of infection with Powassan virus (POWV) was requested by the attending infectious diseases specialist. The CSF sample obtained on admission (4 days after illness onset) was positive for POWV immunoglobulin M, with a POWV-specific neutralizing antibody titer of 32. The child’s seizures were controlled with anticonvulsant therapy with fosphenytoin and levetiracetam, and he was discharged home after 7 days on oral levetiracetam. One month after the onset of symptoms the parents noted that he could no longer sit up unaided, a milestone that he had met prior to the illness. Four months after his illness (aged 10 months), he was reported to have normal motor and verbal development (crawling, walking with a walker, and babbling). He was noted to have a distinct left-handed preference. He was no longer receiving physical or occupational therapy, and was no longer on any antiepileptics. A second MRI performed 4 months after the first revealed gliosis and encephalomalacia in the thalami and basal ganglia bilaterally, with volume loss and evidence of early mineralization in the left basal ganglia.

POWV is a tickborne flavivirus, similar to tickborne encephalitis virus, transmitted by *Ixodes scapularis, I. cookei,* and *I. marxi* ticks (*1*). Transmission of POWV occurs as quickly as 15 minutes after tick attachment (*2*). Clinical presentations of POWV infection range from a febrile illness to severe neurologic disease, with death occurring in approximately 10% of reported cases; long-term sequelae are common (*1*).

During 2006–2015, a median of seven cases of POWV disease (range = 1–12) were reported annually in the United States (*1*). Cases occur predominantly in the Northeast and Great Lakes region; several states (Minnesota, New Hampshire, and Virginia) reported their first POWV disease cases during the last 7 years; it is not known whether this represents spread of the virus within the local tick population, or increased testing and recognition of the virus as a cause of human disease. Despite POWV-infected ticks having been found in Connecticut (*3*), this is the first report of a human case of POWV disease in the state and highlights the importance of considering POWV disease in persons with a clinically compatible illness and obtaining a comprehensive exposure history. Although the illness onset for this patient (early November) was later than might be expected for encephalitis caused by mosquitoborne arboviruses (e.g., West Nile virus, which is more common in the summer months), ticks can be active well before and after peak mosquito season. It is important for clinicians to consider POWV disease whenever a patient in a tick-endemic area is evaluated for encephalitis. Testing for POWV infection is not usually included on arbovirus encephalitis panels and should be requested specifically, as was done in this case. The short attachment time required to transmit POWV highlights the importance of avoiding tick exposure through the use of repellents or wearing permethrin-treated clothing. Tick checks should be performed as soon as possible after exposure, including showering to remove any nonattached ticks, and clothing should be changed afterward.
